# A *Duty* to treat? A *Right* to refrain? Bangladeshi physicians in moral dilemma during COVID-19

**DOI:** 10.1186/s13010-020-00091-6

**Published:** 2020-09-09

**Authors:** Norman K. Swazo, Md. Munir Hossain Talukder, Mohammad Kamrul Ahsan

**Affiliations:** 1 Department of History and Philosophy, North South University, Dhaka, Bangladesh; 2 Department of Philosophy, Jahangirnagar University, Savar, Dhaka, 1342 Bangladesh

**Keywords:** COVID-19, Pandemic, Duty to treat, Medical ethics, Bangladesh, Professional autonomy

## Abstract

**Background:**

Normally, physicians understand they have a duty to treat patients, and they perform accordingly consistent with codes of medical practice, standards of care, and inner moral motivation. In the case of COVID-19 pandemic in a developing country such as Bangladesh, however, the fact is that some physicians decline either to report for duty or to treat patients presenting with COVID-19 symptoms. At issue ethically is whether such medical practitioners are to be automatically disciplined for dereliction of duty and gross negligence; or, on the contrary, such physicians may legitimately claim a professional right of autonomous judgment, on the basis of which professional right they may justifiably decline to treat patients.

**Methods:**

This ethical issue is examined with a view to providing some guidance and recommendations, insofar as the conditions of medical practice in an under-resourced country such as Bangladesh are vastly different from medical practice in an industrialized nation such as the USA. The concept of moral dilemma as discussed by philosopher Michael Shaw Perry and philosopher Immanuel Kant’s views on moral appeal to “emergency” are considered pertinent to sorting through the moral conundrum of medical care during pandemic.

**Results:**

Our analysis allows for conditional physician discretion in the decision to treat COVID-19 patients, i.e., in the absence of personal protective equipment (PPE) combined with claim of duty to family. Physicians are nonetheless expected to provide a minimum of initial clinical assessment and stabilization of a patient before initiating transfer of a patient to a “designated” COVID-19 hospital. The latter is to be done in coordination with the national center control room that can assure admission of a patient to a referral hospital prior to ambulance transport.

**Conclusions:**

The presence of a moral dilemma (i.e., conflict of obligations) in the pandemic situation of clinical care requires institutional authorities to exercise tolerance of individual physician moral decision about the duty to care. Hospital or government authority should respond to such decisions without introducing immediate sanction, such as suspension from all clinical duties or termination of licensure, and instead arrange for alternative clinical duties consistent with routine medical care.

*Noli naturam humanum in te ipso laedere* (Do not injure human nature in yourself)

Kant, *Lectures on Ethics*, 27:347

No one is so fearless or stupid as to discount all risks [1].

Thomas Kirsch MD, MPH (Emergency room physician)

## Introduction

Writing in 2017, emergency medicine physician Cameron Y.S. Lee commented in warning: “The world is due for an infectious disease pandemic of similar proportion as the 1918-1919 Spanish influenza…During such a pandemic where morbidity and mortality are high, do physicians have a duty to treat patients where there are significant risks of contracting the disease that could cause extreme illness and even death to themselves?” [2] As might be expected, Lee answered in the affirmative, with some caveats (i.e., duty based on medical specialty and scope of practice)^1^ [2]. This claim contrasts to survey data of physicians in 2003, according to which “80% of respondents would be willing to continue to care for patients in the event of an outbreak of an unknown but potentially deadly illness, although only 40% said they would be willing to put themselves at risk of contracting a deadly illness to save others’ lives” [3].^2^ Said otherwise, 60% of the respondent physicians would not willingly treat patients when the personal risk of deadly contagion is high. Indeed, during the SARS outbreak, many health care professionals refused to treat patients, despite the argument that “their moral responsibility does not change with changing disease scenarios” [4].

During the MERS outbreak in Saudi Arabia, four consultant rank physicians at Jeddah’s King Fahd Hospital “resigned after refusing to treat patients affected by MERS, apparently out of fear of catching the virus” due to lack of adequate infection control at the hospital [5]. Public response was critical, on claims these physicians had an “unethical attitude” and that “it is a physician’s responsibility to treat patients ‘under any circumstances;’” others allowed for “individual physician choice” consistent with World Medical Association standards, such that “physicians have the right of moral judgment with reference to the interests of various stakeholders that are not exclusive to the physician-patient relationship” [6]. During the Ebola outbreak in Africa, out of 830 health care workers infected, 488 died (59%), many eventually quitting entirely to avoid infection and death [7].

Recently, in the case of Egypt, the Egyptian Medical Syndicate complained of government “negligence” in providing personal protective equipment (PPE) to health care workers and unacceptable clinical operational guidelines as 19 physicians have died amidst 350 COVID-19 infections, the Syndicate decrying the ministry’s “dereliction of duty” as “a crime of killing by irresponsibility” [8, 9]. In Bangladesh, it is reported that out of 2458 physicians having tested positive for COVID-19 as of first week in August, 92 (3.7%) have died, hence the reluctance of some physicians to treat patients suspected of COVID-19 infection, this due to personal risk assessment and not conscientious objection per se as traditionally defined^3^ [10]. Indeed, such reluctance is not surprising in light of one study reporting that frontline health care workers have “more than three times the risk of COVID-19 infection than the general public” [11].

Physician refusal to treat COVID-19 patients should not be surprising, since even the World Health Organization (WHO) guidance on emergency preparedness allows that, while professional codes of ethics stipulate a duty to care, nonetheless, “Health care providers will have to weigh the demands of their professional roles against other competing obligations to assess their own health and to families and friends” [12]. As COVID-19 manifested its virulence and an undetermined global case fatality ratio in early 2020, the question of professional duty has been raised in the public media as a question not to be “glibly dismissed” [13, 14]. Medical professionals in the USA have been engaging the moral questions at issue, with a call for “considered and systematically enacted guidelines for action” [15–17], including “crisis standards of care” [18]. Elsewhere, in Bulgaria, e.g., where there is a “brain-drain” of physicians and higher use of retired doctors, “dozens” of physicians and nurses have simply resigned rather than treat patients [19]. In Russia, a dire shortage of PPE and an incapably bureaucratic management structure have led to a high number of physician deaths amidst a high number of infections among health workers, many continuing to work while infected: “You had no choice,” one physician opined [20]. In Bangladesh, one physician assigned to the care of COVID-19 patients, isolated in hotel while on duty and unable to visit with family during the Muslim Eid al-Fitr holiday under a rule of having to maintain a 14-day quarantine after last working day at hospital, opined, “We joined the job with an oath to serve people and I will try my level best” [21]. Clearly, the duty to treat patients and the duty to family remain in conflict.

It is more or less standard as a matter of professional ethics that a physician who (a) has been granted medical practice and hospital admitting privileges and (b) is on duty at a given hospital, is obligated to provide treatment to a patient who presents at that hospital, whether for inpatient or outpatient medical care. This obligation, understood morally and not legally (juridically), is not without conditions, however. Consider, e.g., that given (1) service specializations (family medicine, OB-GYN, pediatrics, internal medicine, cardiology, intensive care, etc.) and (2) level of clinical practice (senior consultant with board certification, junior consultant, medical officer, senior resident, junior resident) in urban metropolitan (tertiary) hospitals, the scope of a given physician’s permissible and expected medical practice can and often will be limited to those presenting for care in his or her specialty. But, in smaller hospitals, especially in rural areas where the number of medical staff is more limited and the number of beds is minimal, it is not unusual that physicians on staff would rotate in shifts of emergency room (ER) duty but otherwise be expected primarily to treat patients consistent with their areas of specialization and level of clinical training. This is more or less standard for routine medical care in situations characteristic of a range of patients presenting themselves in hospital ERs or outpatient clinics under normal operations.

But, the entirely extraordinary situation of a pandemic involving a novel highly infectious disease [22, 23] such as COVID-19, that has a mode of transmission not fully determined [24], and that presents with symptoms exceedingly variable and atypical, introduces heightened moral conflict among medical practitioners [25–27]. PPE may be entirely lacking or insufficient in quantity relative to rapid surge in patient demand for urgent clinical assessment and care in ERs and intensive care units (ICU). Under these conditions, medical practitioners (especially primary care physicians attending to the usually high daily patient caseload) are hard pressed to respond, especially under conditions of physicians in the clinical setting facing multiple exposures from many patients and multiple opportunities for inoculum with varied virus loads and depending on modalities of treatment. Many face a difficult professional moral decision: (1) Are they to “do their *duty*” and treat a patient suspected of COVID-19 infection, despite lack of or poor quality PPE?^4^ [28] Or, (2) may they, as a matter of professional *right* of autonomous clinical and ethical judgment, “*refrain* from treatment,” appealing, e.g., to the *fact* of inadequate PPE and high personal risk of infection as reasonable justification not to provide medical care under the circumstances of local outbreak, national epidemic, or global pandemic? The answers to these ethical questions are by no means evident even as it is clear that, while physicians grapple with the decision they face “unprecedented stressors” [29, 30]. The public at large must also account for the fact of inadequate hospital preparedness while acknowledging the reciprocal obligations hospital management authorities have “to protect their employees and mitigate risk,” especially among front-line health care workers^5^ [31–33].

Physicians have had to face this moral decision in the best of hospitals in developed countries; and, they normally resolve the moral questions at issue by performing their duty, whatever their reservations about personal risk of infection^6^ [34]. For some this is a matter of inner moral motivation; for others, performance follows from the recognition of prospective legal liability for gross negligence. In a developing country such as Bangladesh, however, this moral question is even more challenging for medical practitioners under local conditions of practice, where:

- PPE safeguards are totally absent in some hospitals and clinics or otherwise dismally inadequate in supply relative to patient demand for urgent infectious disease care; in some facilities PPE are of low quality for actual efficacy under conditions of expected long-term reuse [35];
- ICU beds and isolation wards are limited^7^ [36] (if at all available, not to mention lack of “negative pressure” isolation); in fact, some 45 days since the first confirmed case in Bangladesh in March, reportedly many ICUs were not properly equipped to be operational for the intensive monitoring and delivery of oxygenation required for COVID-19 patients, hence a contributing factor to an early higher death rate relative to recovery rate [37]. And, where ventilation support is available and in use on a given patient in an ICU, a confirmed positive patient may (after initial evaluation) be readily transferred to a government “designated” COVID-19 hospital, on the claim of avoiding admission of a suspected patient who could trigger an uncontrollable nosocomial spread [38]—notwithstanding explicit government directive not to refuse treatment to COVID-19 patients^8^ [39, 40]. Even patients with presentation for otherwise routine acute care (e.g., complications of pregnancy) [41] or emergency care (stroke or heart attack) [42] are declined service in the absence of a certificate of negative test result for COVID-19;
- The volume of patients and protracted length of stay associated with COVID-19 is beyond the capacity of the given hospital to respond. Hospital staff have to balance their response to suspected cases of novel COVID-19 while attending to the usual presentation of patients^9^ [43–45] for infection and disease such as is found in the megacity of Dhaka (population = 20.2 million; population density ~ 48,000 per square kilometer).
- Social stigma [46, 47] of contracting COVID-19 motivates the general public not to disclose possible infection when presenting for medical care and, therefore, place physicians and nurses at risk [48];
- Private hospitals in Dhaka are unwilling to treat patients having (or otherwise suspected of) COVID-19 out of fear of enhanced nosocomial transmission [49], readily informing such patients to present themselves at designated government hospitals.

Triage and transfer of persons suspected of infection (with or without guidelines for fair allocation) [50] under conditions of unavailable systematic COVID-19 testing^10^ [51] are practices that raise questions about the adequacy of clinical decision. For example, an individual suffering from manifest respiratory distress can first present to a nearby medical college hospital, then (for whatever clinical reasons) be transferred to a medical university hospital, then again be transferred to a designated COVID-19 hospital facility on presumption of COVID-19 infection, then be referred again to yet another medical college hospital, and even once again moved to a general city hospital—the extraordinarily extended process of patient transfer eventually contributing to lack of stabilization of the patient’s condition and eventual death [52]. A similar case presented requiring treatment for kidney disease, the patient being declined treatment at several hospitals without test for COVID-19, and eventually dying without proper medical care [53]. Physicians practicing in such circumstances are manifestly at a loss not knowing what they *should* do, both clinically and morally, the shortage of medical and nursing staff and lack of efficacious therapeutics contributing to a perceived medical futility. And, even when they do provide whatever limited clinical care they can in the absence of validated protocols, the mechanism of COVID-19 death is yet unclear, hampering clinical decision-making about appropriate type and scale of interventions if available [54, 55].

In addition to the personal moral decision taken by a medical practitioner in a developing country facing the above situation, there is the further administrative decision to be taken by hospital authority (chief of staff, chief medical officer, etc.) in the event a physician (a) fails to be present for duty or otherwise (b) explicitly refuses to treat patients suspected or confirmed to have COVID-19. Is a physician acting according to professional *right* when s/he chooses not to report for duty or, while present at hospital, explicitly refuses to treat patients suspected or confirmed to have COVID-19? Or, despite the circumstances of inadequate institutional facilities and PPE, as well as minimal training in clinical ethics, is a physician not to be granted reasonable *exception* to an expected professional obligation to provide medical care? Or, are supervising medical or administrative authorities simply to sanction a physician who refuses to perform his or her medical duty, suspending the physician from all clinical practice privileges?

## Case example

According to a report in Bangladesh, a junior consultant in anesthesia and three medical officers failed to report to duty, while a junior consultant in gynecology and a resident physician refused to treat COVID-19 patients, at Kuwait Bangladesh Friendship Government Hospital, a 200-bed hospital in Dhaka that was specifically prepared “exclusively for treatment of patients with coronavirus infection after the outbreak” of COVID-19 [56]. This hospital reportedly has an outpatient department with 50 consultation rooms, OB/GYN with a 32-bed neonatal ICU (NICU), a 22-bed ICU, a 10-bed isolation unit, and a 5-bed high dependency unit (HDU) [57]. Accordingly, all medical staff assigned to this hospital presumably understood that they would be providing services in a hospital that would be treating patients either suspected of or confirmed to have COVID-19, despite their routine outpatient and inpatient shift rotations. The government authority took the decision to suspend these six medical practitioners on the basis of their failure to discharge professional duties, i.e., charging them with negligence and dereliction of duty, the Health Minister warning others who behave similarly of a loss of license to practice medicine. In contrast, a member of the national advisory committee responding to the pandemic and the personal physician to the Prime Minister, Dr. ABM Abdullah, is reported to have commented, “Personal protection is important. Without quality PPE, how will they provide care to a patient? Those who complained over poor quality PPE and masks faced punishment. It is not okay, it is unacceptable, it is injustice… I think the administration should stop harassing doctors.” [58] It was reported the suspension was subsequently withdrawn [59].

The motivations of the suspended practitioners were unclear, although inferences from media reports are that the lack of PPE and associated fear of personal infection were contributing factors, hence not a matter of conscientious objection in the standard sense of that concept. The fear of personal risk is real, of course. Only 420 physicians, most of them junior consultants or medical officers with MBBS degrees only and no specialization, are deployed at hospitals in Dhaka designated for COVID-19 patients [60]. Bangladesh’s early situational context of COVID-19 medical care within 1 month of case detection, as reported by the Bangladesh Doctors Foundation in early April 2020, was that 100 health care workers, including 54 physicians, had been infected, two physicians in ICU [61, 62]. As of the last week of June, the number of infected physicians was reported to be 1341 with 55 deaths [63], the majority as of mid-May reported (*n* = 421, ~ 69%) in Dhaka Division (60% from government hospitals, 35% from private hospitals) [64, 65].

The first physician fatality occurred within 2 weeks of self-imposed isolation and subsequent treatment at a designated COVID-19 hospital in Dhaka, a second physician succumbing on 03 May [66], and two more thereafter [67]. By mid-June it was reported that Bangladesh has the highest physician mortality rate from COVID-19 in the world, at 4% (compared to an “average standard mortality rate in doctors” of 2.5%), around 54 physicians having succumbed to the infection by mid-June [68]. It is entirely problematic for Bangladesh when physicians become infected with COVID-19, because the number of trained physicians is already limited—at around 27,400 [69], a ratio of 0.53 per 1000 national population (compared to a ratio of 2.6 per 1000 in the USA) [70, 71]. WHO reports Bangladesh to have a total of only 35,993 registered/licensed MBBS-level trained physicians for the period 2007–2016, with a “health workforce (doctor, nurse, midwife) density” of 7.4/10,000 population (compared to a recommended density of 44.5/10,000) [72]. There are few specialists in critical care, pulmonology, and intensive care capable of managing ICU patients effectively, the consequence of physicians having lack of experience in such skills including medical mismanagement and negligence in the clinical setting, not to mention failure in infection control procedures, if assigned to such duty involuntarily.

According to WHO, the Bangladesh office of the Director-General of Health Services (DGHS) reported the shortage of PPE stocks, and as of 24 June the DGHS reported a total of 1,268,618 “PPE kits” available [73], although there have been donations of PPE from China and elsewhere and ongoing effort in local production of PPE (but requiring quality control capable of meeting WHO standards). In response to requirements for preparedness, the DGHS “provided training in infection prevention and control (IPC) at hospitals for COVID-19 cases to 710 doctors and 43 nurses; among them two doctors from each district (one residential medical officer and one medical officer from Civil Surgeon office) [73].” This number increased to 915 physicians and 98 nurses as of 05 April, but is clearly insufficient for the purpose of efficacious infection control in the context of COVID-19, hence the high number of COVID-19 infected physicians (*n* = 2458 as of 02 August 2020) and high mortality rate (*n* = 92, 3.8% of confirmed infected physicians as of 02 August 2020). These data are consistent with a recent study showing that the risk of COVID-19 infection is greatest among front-line health care workers in in-patient settings where PPE reuse practices were prominent—“Compared with the general community [in UK and USA], front-line health-care workers had a twelvefold increase in risk of a positive test after multivariable adjustment (adjusted HR [hazard ratio] 11.61, 95% CI 10.93-12.33...)” [74].

## Assumed duty to treat

There have been emergent infectious diseases in recent decades, such as SARS-1 (2003), H5N1 avian influenza (2003), H1N1 influenza (2009), MERS (2012), avian influenza A H7N9 (2013) and Ebola (2014–2016), and that have engaged governments in the task of planned preparedness as well as individual medical practitioners in the task of providing “front-line” medical care. One survey of employee perspectives has shown that most workers (60%) believe it is unethical to abandon the workplace during a pandemic, in view of a “duty to care,” while 65% desired “autonomy to decide whether or not to work,” although “79% would agree to volunteer, given some incentives and protection options, the most salient being protective equipment (with relative training for use) and infectious disease training” [75]. These responses are important for the fact that current medical practice specializations are such that most practitioners are not trained to manage infectious disease or to do so under infection control protocols that are established in situations of epidemic or pandemic. They are important also for the fact of a recognized duty to provide care under the extraordinary situation of pandemic while preserving the right of autonomous choice that then registers as “volunteering” to provide due care.

Heidi Malm et al., [76] writing in 2008 after public health experience of SARS, provide one example among many papers in recent years that engage the bioethical question of a duty to treat during a pandemic [77, 78]. The authors examine various ethical positions that ostensibly warrant a duty to treat (“expressed consent, implied consent, special training, reciprocity (also called the *social contract view*), and professional oaths and codes”). These moral warrants are understood to apply generally to physician practice, in which case the context of medical practice (urban/rural; developed country/developing country; board certified consultant/junior resident, etc.) is ostensibly irrelevant. Public health emergencies, whether due to pandemic or natural disaster, are understood to entail restrictions on liberty consistent with public health mandates, hence even restrictions on professional practice and thus the personal decision-making of medical practitioners [79].

Yet, experience with SARS in 2002/2003 showed health care workers suffering significant infection while on duty (40% of cases in Toronto; 18% of cases in Taiwan; 25% of cases in Hong Kong) [79]. Uncertainties concerning COVID-19 disease progression but related to SARS-1 contribute to medical practitioners being reluctant to provide care when otherwise duty would be performed. Malm et al., conclude that, “none of the defenses [of these various moral warrants] is currently sufficient to ground the kind of duty that would be needed in a pandemic” [76]. But, then, this claim is all the more pertinent in the case of physicians practicing in a developing country where institutional capacity is woefully inadequate for infection control. Physicians in these cases are expected not only to provide medical care to a given patient presenting with suspected or confirmed infectious disease but also to exercise due care to avoid further transmission (exposure to themselves and transmission to other patients) within the hospital setting. Thus, their “medical skill” is not limited to treating a given patient, but also and importantly to contributing to infection control, working to avoid the spread of nosocomial infection but also community spread, especially when this is a matter of highly pathogenic transmission. Thus, the “scope of duty” is at issue when one is expected to practice outside his or her medical specialty under the press of urgent care.

Writing in 2018, David Orentlicher argued that, “restoring a strong duty to treat would protect patient welfare without subjecting physicians to undue health risks” [80]. Here, e.g., one may account for the general principle of beneficence, according to which physicians are expected to perform their duty “to the best of their ability” even as they avoid intentional or negligent harm to those they treat (the principle of non-maleficence). But, clearly, physician “ability” will (and does) vary consistent with medical specialty so that, e.g., not every medical practitioner can be an emergency room physician dealing with severe respiratory distress such as is clinically present in many COVID-19 patients or an intensivist managing such patients in ICUs or in isolation wards. Orentlicher also argued that, “it is counterintuitive to see a weakening of the duty to treat in an era when advances in medicine make it much more likely that physicians can provide effective care to affected patients and much less likely that physicians will themselves succumb to a new public health pandemic” [80].

The utility argument here is that the social benefit in patient care exceeds the personal risk to the physician, hence a strong duty to treat despite disinclination. Jeremy Bentham’s act utilitarianism upholds this view, although J.S. Mill’s utilitarianism does not share this view^11^ [81]. This kind of probability or benefit/risk ratio may hold for medical practice in the USA in most health care facilities, but this is certainly not so for medical practice in a developing country such as Bangladesh where most medical care is a function of public sector (i.e., government) infrastructure rather than private medical practice and associated hospital facilities. On the contrary, in a country such as Bangladesh physicians are quite likely to succumb to a clearly insidious SARS-COV2 virus with lethal COVID-19 infection, precisely because so-called “advances in medicine”—PPE with adequate training in their use and infection control practices, patient diagnostic and treatment options (radiographic imaging, wall/piped or cylinder^12^ [82, 83] or concentrator-supplied or high-flow oxygenation, non-invasive ventilation using continuous positive airway pressure (CPAP) therapy, high compliance pulmonary management with mechanical ventilation (existing protocols for which are in question [84, 85]), antiviral therapeutics, steroids, ACE-inhibitor (especially for IL-6 cytokine response manifest with COVID-19), and angiotensin receptor blocker drugs, etc.—are all too often severely limited as resources where these physicians practice, including sorely limited options for institutional quarantine or isolation. This is not merely hypothetical; it is a matter of fact, a fact that is an operational foil to any prima facie appeal to a strong duty to treat.

Howard Markel’s views in the context of Ebola are pertinent to the special case of a developing country response and assessment of physician responsibility [86]. Markel reports that during the Ebola public health emergency in West Africa, “relief and humanitarian organizations urged the doctors, nurses, and other health professionals working for them to flee ‘the hot zone’ and go home.” Many would find such counsel morally illicit under the circumstances if a strong obligation to provide medical care is assumed. But, with novel SARS-COV-2 and the unpredictable and precarious progression of COVID-19 even under the best of available inpatient resources, the question Markel raises remains front and center: “How should one care for patients during an epidemic disease that modern medicine has not yet figured out how to effectively treat, especially when the disease in question holds a very real risk of killing both the patient and the health professional?” [86] It is the latter prospect that presents the dire moral dilemma for the medical practitioner in the context of a developing country institutional capacity to respond.

Hence, it is important to acknowledge that medical practitioners face genuine moral dilemmas insofar as they have conflicting moral obligations—being husbands, wives, parents, etc., not to mention their duties not only to the present but to preserve their own health to be able to provide care to a near-future generation of patients as the current crisis resolves [13]. A.K. Simonds and D.K. Sokol summarized the main competing duties thus: “1) duty to patients; 2) a duty to protect oneself from undue risk of harm; 3) a duty to one’s family; 4) a duty to colleagues whose workloads and risk of harm will increase in one’s absence; and 5) a duty to society” [79]. How to choose among competing duties has no automatic a priori algorithm and is mostly situational and depends on voluntary assent to ethical guidelines^13^ [87].

American cardiologist Sandeep Jauhar, writing during the current COVID-19 spread in New York city, reminded that, “The ethics manual of the American College of Physicians, for example, states that ‘the ethical imperative for physicians to provide care’ overrides ‘the risk to the treating physician, even during pandemics” [13]. The Infectious Diseases Society of America likewise insists on a duty to treat “even at the risk of contracting a patient’s disease” [13]. This is a position consonant with that of the American Medical Association, which holds that physicians have a duty to treat “even in the face of greater than usual risks to physicians’ own safety, health, or life” [13]. A similar guideline is given in the UK General Medical Council’s Good Medical Practice, which privileges a duty to treat over risk to the physician [79]. Yet, as Jauhar reminds, in the case of a SARS outbreak in Toronto, Canada in 2003, “in which nearly half of the infected were health professionals, many health care workers refused to show up at their jobs”—not surprising in a situation where ICU staff intervened with intubation and mechanical ventilation, procedures enhancing aerosolized transmission of the virus. Thus, Jauhar queries: “How do we balance our professional and personal obligations?” [13].

Clearly, if one accepts the American medical ethic to be more or less standard such that it applies in principle to how Bangladeshi doctors perform their duties, then absence of or limited PPE and personal risk of infection are no bases for refusal to treat COVID-19 patients in Dhaka. On the other hand, as with SARS in 2003, there is ample reason to “set professional duty alongside other individual commitments and broader social values” when judging physician duty to treat [88]. Reasoning on this basis, Howard Brody and Eric Avery concluded, however, “A solid ethical basis for the health professional’s duty to treat the victims of emerging infectious diseases, even at some level of personal risk, has proven elusive…In sum, we have discovered no *single* ethical foundation for a duty to treat that would be commensurate with the needs posed by an emerging infectious disease pandemic” [88]. The example of health care workers in Toronto illustrates that physicians and allied health care workers insist on autonomous decision under circumstances of pandemic and high infection among such professionals. Bangladeshi doctors could, with good reason, appeal to such an example to warrant their own refusal to show up for duty and/or to refuse to treat patients. Hence, one such as Jauhar reasonably counsels and concludes: “Doctors and nurses and other health care workers may be heroes in this pandemic, but we will not be martyrs” [13]. The point has consonant commentary in Bangladesh, as attorney Rashna Imam writes: “We need our health-care workers more than ever, but that does not give us the right to make inhumane demands of them that may be tantamount to human rights violations. Moreover, given the dire shortage of healthcare workers in Bangladesh, even if they were to voluntarily embrace martyrdom, it would be disastrous for us in the long run”^14^ [89].

While many in the American public and throughout the world characterize health workers to be brave and heroic during the pandemic, there should not be a false dichotomy imposed such that they should be acclaimed either heroes or martyrs on the one hand or lacking in moral fortitude on the other hand. The clinical and professional ethics at issue are not so simplistically settled as if this were a choice a physician, nurse, etc., should intentionally make whatever the circumstances of local institutional capacity. Rules present in otherwise standard operating procedures are for the most part inapplicable. As New York emergency room physician Dr. Helen Ouyang relates from her experience, “coronavirus is lawless. It obeys no rules” [90]. Hence, one cannot depend on thinking of “perfect” solutions, not even “good” solutions—“Better to be lucky,” she says. As an ICU physician in Italy put it, “…it’s impossible to work. And there is no space for imagination during humanitarian crisis. If you use a lot for the first patient, then you have no treatment for the next patient. You have to reorganize everything. You have to reorganize your mind; you have to reorganize your work; you have to reorganize your personnel and health care people” [90]. The COVID-19 pandemic creates a gestalt-type shift in the ethical question, precisely because mental health professionals “worry that doctors will sustain moral injury from having to allocate medical equipment and care” [90, 91], decisions that are more forced by the press of urgency than by any meaningful rationality.^15^ Moral injury^16^ [92–94] is reflected in the question Ouyang puts forward: “Is this how the dead leave the world now?” [90].

## Settling the moral dilemma

Medical practitioners consider themselves autonomous moral agents capable of self-determination relative to a range of personal and professional interests, despite institutional and professional ethical code constraints and restraints upon their conduct. Physicians may or may not adopt a “principled” approach to moral decision, accounting for the usual moral principles of *autonomy* (which here has to be construed as both patient autonomy to consent to medical care interventions and also physician autonomy to make a professional decision on some moral basis), *non-maleficence, beneficence*, and *justice* as fairness. However, as Simonds and Sokol opine, these principles do not “translate into specific action-guiding practice” without interpretive application; indeed, they assert, “It is naïve to think that a universal, practical algorithm can be derived from the principles” [79].

Accordingly, *situational* clinical practice may be engaged and resolved ethically on the basis of appeal to typically “probabalist,” hence defeasible, reasoning associated with a set of rules proper to *casuistry* [95]. Casuistry, as recommended by Albert R. Jonsen, allows the clinician to account for: (1) the “morphology” of a case, i.e., “the invariant structure of the particular case whatever its contingent features,” but also “the invariant forms of argument relevant to any case of the same sort;” (2) “taxonomy,” i.e., situating “the instant case in a series of similar cases, allowing the similarities and differences between an instant case and a paradigm case to dictate the moral judgment about the instant case,” judgment being such that the clinician accounts for the ways in which “circumstances and maxims appear in the morphology of the case itself and in comparison with other cases;” and (3) “kinetics,” i.e., understanding how “one case imparts a kind of moral movement to other cases,” given that “different and sometimes unprecedented circumstances may move certain marginal or exceptional cases to the level of paradigm cases” [95].

Writing elsewhere about physician practice in the context of epidemics, Jonsen et al. make the important point that in this situation “many of the sick and potentially sick are not ‘patients’ of individual physicians” [95]. Consistent with current COVID-19 clinical case presentations, emergency room physicians, intensivists, critical care specialists, pulmonary care specialists, infectious disease specialists, etc., are all called to contribute together to managing patients in emergency rooms and ICUs, even as medical staff shift rotations mean that a given patient does not have a dedicated physician responsible for inpatient care in the usual sense. Jonsen et al., comment that while physicians have “long accepted that infection from their patients and work setting is an occupational risk” and that, accordingly, they “are aware that precautions must be taken,” nonetheless “At the same time, the duty to preserve health and protect family, with the corresponding right to do so, is legitimate” [96]. Recognizing as much, Jonsen et al., recommend: “The extent of this duty must be evaluated with respect to the nature, probability, and seriousness of the risks, alternative strategies, the infringement on others’ rights, and the social consequences of various courses of action” [96]. This view clearly contrasts to one such as proposed by Chalmers C. Clarke, who identifies a duty to treat by appeal to facts of “covenant, consent, contract, compensation, and capability,” medical skill placing physicians in the position of “social lifeguards, especially during times of critical medical need” [87].

Thus, in the case of COVID-19, it is already well-known that the risk of infection for physicians in emergency rooms and ICUs is high due to the high volume of patients presenting and the opportunity for transmission within such settings due to limited isolation and interventions such as intubation and mechanical ventilation that add to aerosol spread of the virus. “Prophylactic measures” with PPE are likewise constrained due to shortage of supplies within a given hospital, some having to re-use single-use PPE throughout an entire shift while moving from patient to patient. To the extent a given medical practitioner is aware of applicable codes of ethics, s/he may know that the American Medical Association Ethical and Judicial Council directs that, “A physician may not ethically refuse to treat a patient whose condition is within the physician’s realm of competence,” without appeal to either fear or prejudice, when the clinical evidence is that a patient is positive for infectious disease [87]. Thus, the authors argue, while crises of infectious disease challenge “the moral standing” of health professionals, “The public expectation that health professionals freely accept responsibilities is strong. The general principles of social justice emphasize that those who have certain skills should share them for the public good” [87]. Hence, on this view the imperatives of social justice trump appeals to individual physician autonomy even during situations of epidemic.

One might, of course, also appeal to the authority of a model of moral decision such as proposed by James Rest, i.e., (1) having *moral sensitivity*, “the ability to see an ethical dilemma, including how our actions affect others,” (2) *moral judgment*, “the ability to reason correctly about what ‘ought’ to be done in a specific situation,” (3) *moral motivation*, “a personal commitment to moral action, accepting responsibility for the outcome,” (4) *moral character*, “courageous persistence in spite of fatigue or temptations to take the easy way out” [97, 98]. These are all traits one may reasonably expect a physician to possess as s/he works to resolve moral quandaries, even in the bewildering situation of the COVID-19 pandemic. But, the fact is that not all physicians have had or will have the opportunity to think about such features of moral decision and prefer to defer to stipulated protocol, which account for prior medical experience and clinical precedent provided from similar disease therapeutic regimens (e.g., in the case of COVID-19, assuming ARDS protocol apply) though they may not, in which case any given physician may object to following protocol when the immediate clinical evidence questions this [99–103]. There are additional reasons to question casuistic (i.e., rule governed) approach to morality, including (a) the requirement of continual growth of rules to cover new situations,^17^ (b) the requirement of rules for application of rules,^18^ (c) the creation of a false sense of morality through meeting minimal requirements,^19^ and (d) encouragement to search for loopholes^20^ [104].

In contrast to such approaches, experimental moral psychology examining individual decision-making explains that, “Moral judgments and decisions are often driven by automatic, affective responses, rather than explicit reasoning” [105], a fact which does not exclude the moral decisions of medical practitioners. Indeed, even when such reasoning is examined ex post facto, individuals are unlikely to change their minds on the initial decision taken [106]. Such experimental results are consonant with other studies published by neurocognitive psychologists such as Joshua Greene and Jonathan Haidt [107–109]. Yet, consistent with the general logical dictum not to commit the naturalistic fallacy, i.e., deducing “ought” from “is,” moral philosophers do not take up such empirical results to be normatively controlling and instead work with various models of moral reasoning even as they recognize the force of emotion and motivations of inclination and self-interest in relation to expectations of moral duty.

### Considering a duty to treat

What professional duties should physicians have during a pandemic? Are these the same as in the normal situation of medical practice? Should physicians continue their service where there is a considerable risk of life due to unavailable safety equipment, challenging work environment, blurry guidelines, and insufficient expert knowledge? Are there any *special* duties for physicians in a pandemic? If a physician refrains from duties in a pandemic, for what is ostensibly a justified cause, is it medical malpractice, negligence of duty, or an exercise of individual autonomy? What types of institutional measures are justified in this case?

There is no straightforward way to answer these questions. The context and specific cases are relevant here. In general, professional guidelines for physicians direct their duty, commitment to service, and their relation to patients’ welfare. The Hippocratic Oath, central to traditional medical codes of ethics, requires of a physician a commitment: “I will use…my greatest ability and judgment, and I will do no harm or injustice to them” [110]. Is it not an injustice to patients, therefore, when a physician refrains from duty in a pandemic? The American Medical Association (AMA) Code of Medical Ethics, Opinion 8.3, clearly states the physicians’ obligation even in a pandemic: “First and foremost is the obligation to ‘provide urgent medical care during disasters’, an obligation that holds ‘even in the face of greater than usual risk to physicians’ own safety, health or life” [111].

These professional codes should be the subject matter of medical colleges’ curriculum so that medical students can prepare themselves for their greater role as physicians in future. Orentlicher argued that the physician’s role is not exceptional compared to other service providers (police officers, firefighters). He notes, “when medical students embark on their careers, they understand the risks that they will face…[The] occupational risks for physicians are by no means exceptional” [80]. Orentlicher observes that the professional risks for physicians are increasingly diminished; and, “a strong duty to treat ensures that patient needs will be met, and such a duty would not subject physicians to undue health risks” [80].

Unfortunately, in Bangladesh there is a significant lack of medical ethics training in the MBBS curriculum. The Curriculum for Undergraduate Medical Education (2012) incorporates some discussions on medical ethics, health ethics under forensic medicine and community medicine, along with some other subjects, but allocates a few hours of teaching on these, which may not be sufficient to address moral dilemmas [112]. A good number of scholars have suggested that implementing ethics and moral education in medical curricula and arranging short training on it are also a part of pandemic emergency preparedness. T.C. Voo and B. Capps, e.g., suggest, “the medical curriculum will benefit, with students being educated in the duty of care and its legitimate expectations, as well as other ethical issues that take centre stage in a pandemic response” [113].

One may argue that physicians in Bangladesh should be inspired more from moral guidelines and can thereby continue their service for many vulnerable helpless patients. It seems that only for some *extraordinary* cases (e.g., the physician presumptively infected, given unconfirmed symptoms; his or her family members already infected; presence of other acute physical and/or mental problems, whether his/hers or family; local social disorder or unrest due to pandemic; and so forth) a physician can have a right to refrain from medical practice in a pandemic. In a developing country like Bangladesh, however, there are some reasons why this exception is not generally justified:

First, the country with scarce resources has invested public funds significantly to produce a physician (from primary and secondary schooling to completion of medical study). Citizens, therefore, have a claim to reasonable return on that investment, particularly in a critical moment such as a pandemic.

Second, the choice of medical profession was an informed choice for a medical practitioner who is born, reared, and lives in the country. So, to refrain generally from the duty to treat would mean a violation of professional oath and the “promissory note” tacitly or explicitly accepted during medical education.

Third, and more importantly, treating patients in Bangladesh (or any developing country) during a pandemic is not just a *mere* duty. Rather, it is a priceless service, even a *sacred* duty, a moral commitment of the physician to the *nation* that formed him or her as a professional. S/he serves not merely a given patient but rather serves the whole nation, especially in the midst of a pandemic.

Hence, when many world leaders are observing that their countries are “at war” and fighting against an “invisible enemy,” physicians and healthcare providers are “soldiers” in that war. As in actual war, a soldier ought not refrain himself or herself from a duty to engage the enemy without any justified reason. Similarly, a physician ought not exercise his or her right to refrain from service in a pandemic. In war, we see many “heroes” being seriously wounded who yet continue to perform their duty, motivated, e.g., “to save the motherland.” A physician may be expected to act in parallel to save the people during a pandemic. Thus, one may affirm that there are some issues (such as crisis management skills, having true and reliable information about the local pandemic situation, having effective communications, having safety measures in place, and government support) that need to be considered and which may affect whether a physician has a manifest duty to treat or a right to refrain under the circumstances. If the physician’s claim is that there is a *right* to refrain from the duty to treat, then it should be exercised only on a plea of extraordinary conditions of medical practice.

That said, however, the duty of a physician in a pandemic might not mean just providing treatment. There is no standard treatment regimen or established effective therapeutic for COVID-19 disease. It could rather mean many other important things, for example, providing mental support, sharing a caring and respectful attitude, intimation of humane empathy/sympathy, prompt responsiveness to patients, cordial communications, kindness, professionalism, and so forth. Furthermore, the fact is that all physicians need not perform the same type of duty, accounting for level of experience and specialty. Cameron Y.S. Lee, e.g., remarks, “physicians who specialize in the treatment of infectious diseases, emergency medicine, and anesthesia would be critically needed in such a pandemic,” and it is understood they would be more so “directly exposed to patients infected with the contagious virus” [2]. Thus, such specialization heightens the fact of a duty to treat, in contrast to a primary care practitioner who has no such skills.

Overall, one may expect that, especially in a pandemic, a physician’s dedication to medical practice contributes to achievement of a virtuous character trait, viz., trust. A trustworthy physician is an asset for any country, but it is very much expected in developing countries like Bangladesh. The UK’s General Medical Council Guidance “The Duties of a Doctor Registered with the GMC,” e.g., states, “patients must be able to trust doctors with their lives and health” [114]. The moral inspiration as reflected in this and other medical codes is towards becoming a “good doctor.” In explaining “professionalism in action” the GMC adds, “patients need good doctors. Good doctors make the care of their patients their first concern” [114].

Of course, it is obvious that medical practice in Bangladesh is challenging. Physicians struggle with a huge number of patients on a daily basis, and so they engage the patient in very limited time; they have a poor working environment; they are faced with high illiteracy of patients; medical equipment is scarce; and so forth. Their role as practitioner becomes even more challenging in a pandemic due to expected unlimited working hours, in which case they become tired, stressed, exhausted, sleepless, feeling constantly unsafe and at high risk due to incessant exposure to a readily transmissible contagion. Under these circumstances of extraordinary clinical practice, if any physician believes him/herself unable to perform a normally expected duty, then s/he should not be forced to do so. The authoritative ground for exception could be review and approval from a hospital ethics committee (in contrast to, e.g., hospital administrative officials or chief medical officers acting without supplemental deliberative ethics review). This assumes a decision issued by such a committee works to deliver a judgment consistent with justice as fairness. The WHO interim guidance for COVID-19 stipulates, “allow[ing] health workers to exercise the right to remove themselves from a work situation that they have reasonable justification [to do so]” [115]. This interim guidance is applicable in the context of Bangladesh’s response to local epidemic of COVID-19.

### Kantian consideration of a physician’s duty to care in an emergency

#### Duty to care

As mentioned earlier concerning duty to care, above and beyond the codes of ethics^21^ [116] there are specific guidelines that offer general direction for practitioners and healthcare professionals^22^ [117], one of which is the ‘four principles’ approach^23^ developed by Tom Beauchamp and James Childress. ‘Duty to care’ is understood to mean a practitioner is obligated to treat patients under routine, everyday circumstances. The controversy starts when routine is changed to crisis, with its associated uncertainties, as in the context of the COVID-19 pandemic. When some physicians refuse to provide medical care, appealing to personal risks as the reason, the discussion of limits to the presumed duty to care begins. Daniel K. Sokol comments that the grave difficulty here is of an unambiguous definition of duty to care [118]. There are no preset boundaries or limits to this duty, and it is subject to individual interpretation^24^ [119]. Ethicists, therefore, face a challenge to explain a practitioner’s duty towards patients.

For some^25^ [119], one of the duties is to ensure the obligation of beneficence towards patients, and to do so despite the inherent dangers of exposure to contagion associated with close physical contact. By entering into the profession, a practitioner agrees not only to abide by new rules, but also to accept dangers that would be unacceptable to most. This duty may vary among different specialties of medicine^26^ [119]. Some, however, do not share this view and argue that professional obligations are conditional. Dr. Sandeep Jauhar opines,

I believe health care workers will continue to make the sacrifices necessary to treat patients. However, it would be a mistake for people to assume that our professional obligations are u5nconditional. An unconditional obligation would absolve society of its own responsibilities. And there are many. For instance, health care workers should not be forced to incur additional risk because people don’t want to practice social distancing (vacationers flocking to Florida beaches during spring break come to mind) [13].

This implies that the moral controversy starts when routine converts to crisis. Immanuel Kant’s ethics of duty are pertinent in this context, specifically to the end of assisting to surmount the moral dilemma in question.

#### The Kantian standard approach to duty to care

In his *Groundwork for the Metaphysics of Morals,* Kant instructs that there is a realm of laws applying to our behavior, hence morality. The aim of *Groundwork* is “the identification and corroboration of *the supreme principle of morality*” (4:392) [120], which Kant calls the “categorical imperative.” In the most general sense, this moral principle requires us to act on those principles that are laws in themselves. In addition to respect for humanity, this principle represents itself as an expression of the human capability for autonomy and self-governance. It is a truth of reason and, therefore, all rational creatures are bound by this principle. Kant continues:

There is, therefore, only a single categorical imperative and it is this: *Act only according to that maxim through which you can at the same time will that it should become a universal law principle that you can will to become a universal law* (G 4:421)^27^ [120].

Consider that at the time of his or her graduation from medical school, a physician who is trained in the treatment of infectious diseases takes an oath to treat patients. Some may argue that a physician who refuses to treat a patient suffering from a contagious disease like COVID-19 violates his oath thereby. Thus, the physician’s action is logically inconsistent with his or her professed duty, and thus fails a test of practical rationality such as Kant prescribes. Taking this professional duty to be grounded in the categorical imperative of universal moral law (the first formulation of categorical imperative, i.e., the principle of universality), one may argue that the physician does not act from a motive of duty that is expected by universal moral law, and hence s/he acts immorally.

Kant’s second formulation of the categorical imperative states that people should not be treated merely as a means to some end because nobody would admit as valid any purpose for an action directed at themselves that is morally improper. It suggests that a physician should treat her/his patients who have been suffering from a pandemic disease in the same way that (s)he desires to be treated if (s)he (her)himself were a patient suffering from the same pandemic disease. Thus, if a physician refuses to treat patients, in effect treating others as a mere means, this fails the spirit of the second formulation (where Kant holds that rational beings both give and need respect on a mutual and equal basis).

The third formulation of Kant’s categorical imperative states that rational beings jointly constitute a community of agents who can accept only those laws that they have given to themselves. If a physician upholds the principle that (s)he will perform a duty to patients when and where (s)he judges it to be clinically warranted, but avoids this duty when (s)he perceives a personal risk of contagion, then this would not be morally acceptable. For, (a) there would be no one to treat patients whenever a pandemic occurs and (b) public trust in physicians would be reduced as the public at large realizes that physicians may withdraw the moment the danger of contagion reaches an alarming level.

A number of questions arise: “Is the Kantian guideline, as given in his *Groundwork*, applicable to medical practitioners performing their duty to patients presenting with COVID-19?” Or, “does the sentiment of practitioners in the face of contagion exempt them from their duty to treat these patients?” It seems that the moral principle implies a uniform duty of care for all physicians under all circumstances. It seems also that Kant’s principle does not permit variations of a limit to the duty to care, e.g., according to changing scenarios of infection and disease. Appeal to personal interest is not morally compelling insofar as this does not accord with duty. In short, the fact of a novel disease such as COVID-19 does not diminish the responsibilities (e.g., duty of beneficence) physicians have to patients. Hence, it follows that practitioners are morally culpable if they disengage from care due to COVID-19 patients.

#### Kantian consideration of practitioners’ duty in an emergency: the case of Bangladesh

Ron Kaczorowski writes that, “across many levels of the healthcare community, COVID-19 has thrown some significant challenges towards traditional thinking and approaches, which must be addressed promptly—with the understanding that what was true just yesterday may not be true today … or even tomorrow” [121]. Many ethicists have contended that normative moral theories that make disproportionate demands should be rejected or otherwise be markedly revised [122–124]. This is the idea of “*demandingness objections*.”^28^ The demandingness objection is usually discussed as a problem for consequentialism,^29^ but there is also an emerging debate focusing on Kantian ethics which is criticized for the same reason.

The question arises: “Is this criticism relevant to a Kantian ‘ethics of duty’ as a whole?” In his *Lectures on Ethics*^30^ [125], Kant elucidates the notion of emergency and explains why one may act differently in an emergency. There is no doubt that the global pandemic has created a global public health emergency with physicians having no clear clinical protocols to guide them. Accordingly, one may clarify Kant’s understanding of emergency and consider its relevance in present context.

Kant begins his analysis of the notion with the claim that “whoever may have told me a lie, I do him no wrong if I lie to him in return, but I violate the right of mankind; for I have acted contrary to the condition, and the means, under which a society of men can come about, and thus contrary to the right of humanity” [124, 125]. Kant then argues, however, that if, in all cases, we were to continue to be faithful to every detail of the truth, we might often expose ourselves to the mischief of others, who seek to manipulate our truthfulness to sordid ends:

…since men are malicious, it is true that we often court danger by punctilious observance of the truth, and hence has arisen the concept of the necessary lie, which is a very critical point for the moral philosopher. This generates an ethical quandary. For seeing that one may steal, kill or cheat from necessity, the case of emergency subverts the whole of morality, since if that is the plea, it rests upon everyone to judge whether he deems it an emergency or not; and since the ground here is not determined, as to where emergency arises, the moral rules are not certain (27:449) [125].

Thus, it is important to note that if one’s bona fide plea in the context of a moral dilemma is that one faces an emergency, then, for Kant, the usual rule of a physician’s duty to treat is *subverted*. Since the moral ground of the action in the case of emergency is not determined a priori, then the applicable moral rules are not certain, and, where not certain, morality cannot be applied strictly. Individual discretion is, therefore, permissible without imparting guilt for someone having violated a strict rule of morality.

The impression here is that Kant isolates a quite special ethic for emergencies from the more commonplace moral perspective for which he insists on a threshold deontology. To be more specific, for Kant, a necessary lie may occur if the plea is to a condition of emergency. A special ethic for emergencies entails that “one has done wrong in doing what is nonetheless right” [126]. That is, as Bernard Williams puts it, one does what is “normally morally wrong but morally permissible in certain circumstances” [127]. This then raises the question of self-regarding duties in relation to duties to others, and Kant’s distinction of perfect duties^31^ and imperfect duties.^32^

Perfect duties to self, such as the prohibition of lying and suicide, specify concrete actions from which we ought to refrain anyway. Perfect duties to others can be duties of right (that follow from rights others have) and thus be legislated into positive law and externally enforced (VI: 232.1–29) [125]. Imperfect duties to self (such as self-perfection or self-improvement) and imperfect duties (such as to help others or beneficence) instruct agents to incorporate certain obligatory ends into their maxims. Imperfect duties are conditional in the sense that they are to be exercised only on condition that no perfect duty would be violated by their exercise. The reason for this, according to Kant, is that violations of imperfect duties when universalized generate only a contradiction in what one wills, whereas violations of perfect duties when universalized generate a strong contradiction in thought. Perfect duties are typically negative and, hence, more than one of them can be fulfilled simultaneously. Imperfect duties always must give way to perfect duties, and they do not directly conflict with each other in concrete situations, since they do not require specific actions, but only a commitment to ends; and, an agent can be committed to more than one end at the same time [128].

In short, perfect duties always trump imperfect duties in the sense that we may never violate a perfect duty in order to further an obligatory end. To be more specific, as Kant claims (27:341) [125], self-regarding duties actually take first place and are the most important of all duties. Nevertheless, special obligations matter for delineating what particularly we owe and to whom we owe it. And, this substantially reduces the “demandingness” otherwise expressed in the Kantian view. In support of this, M. van Ackeren and M. Sticker argue that there is one kind of perfect duty that can moderate demandingness more than other perfect and imperfect duties, namely, a duty of right towards one’s own children [128]. For them, in the Kantian system of duties the demandingness of the duty of beneficence may be “internally” moderated, meaning a special obligation allows one to attach greater moral significance to those near and dear [129, 130]. Special obligations specify our general duty of beneficence, and they moderate our imperfect duties to strangers.

In light of the above distinctions of duty, one asks: (1) May physicians appeal to different limits of duty to care for patients because they have a special imperfect duty of right towards their own family? (2) Should physicians practicing in the context of Bangladesh have different limits of duty to care than medical practitioners in a developed country, appealing to the fact that physicians in Bangladesh have a higher chance of exposure to a pandemic due to inadequacy of overall health care services and infrastructure for pandemic preparedness? Kant’s recommendations on duties in an emergency have a significant implication in answering these questions. Physicians have a special obligation to family members that diminishes the demandingness of the duty of beneficence and moderates duties they have to COVID-19 patients. The “lawless” clinical features of the disease, local institutional incapacity for proper treatment, and the absence of adequate and quality equipment including PPE make the clinical situation (a) nearly suicidal for physicians and (b) a context of negligent harm to other patients. On this line of reasoning, providing medical care to COVID-19 patients under this set of emergency circumstances is a violation of a perfect duty to self-protection. Hence, imperfect duties to treat COVID-19 patients are not unconditional for physicians anywhere in the world, thus including Bangladesh. The appeal to emergency stands out as a significant factor in balancing different obligatory ends, e.g., between duties physicians have to their own family members, to themselves, and to COVID-19 patients. Thus, a Kantian system of duties warrants physicians internally minimizing the demandingness of their duty of beneficence in a situation of pandemic emergency.

In his *Lectures on Ethics* (27:67) Kant commented also on moral sympathy. Motivated by moral sympathy with a patient having a contagious disease, and ignoring the risk of losing her/his life, a physician may well decide to treat. But, that is just a “sacrifice” out of rectitude, for (s)he is not obliged to do that [125]. Kant maintains the view that “there are moral rules of obligation which do not, however, bind, for example, to help a person in distress” (27:259) [125]. For him, one may provide assistance, but not to one’s detriment (27:260) [125]. Also, Kant argues that, “A person can indeed serve as a means for others, by his work, for example, but in such a way that he does not cease to exist as a person and an end” (27:343) [125]. What is more, if a physician, as a matter of public interest, chooses to treat patients under conditions of highly contagious pandemic and, in doing so, (s)he loses her/his life, then Kant would say, (s)he has lost her/his life to *fate*, not to a *free choice* that amounted to suicide. Rather, s/he has given up life “in order to have lived in an honourable way (27:377–378) [125].”

However, assessing the Kantian recommendation, Ackeren and Sticker [128] argue that Kant does not say much about finding the right balance between the obligation to help persons in distress, e.g., patients, and special obligations towards family. For the authors, special obligation can be of only limited moderating force to reduce a physician’s duty of beneficence. Although personal relationships entail special obligations, e.g., to one’s own children, the majority of personal relationships (such as friendship, relation to parents, benefactors, etc.,) do not do the same. They argue that special obligations are powerful sources of moderation in many mundane, or non-emergency, cases. An agent might have to trade in very different currencies, e.g., emotional comfort of those near and dear vs. the suffering and possible death of many patients. The authors conclude:

It is safe to say that the Kantian system of duties does not contain any general prescriptions implying that our special obligations to family, friends and benefactors who are not in an emergency situation and insofar as they do not issue in duties of right substantially reduce what we have to do for those in life threatening situations, and the moral salience of emergency speaks against such a reduction. The moral salience of emergency, however, leaves at least one form of moderation open, namely, as a tie-breaker in cases of emergencies to loved ones vs. emergency to strangers [e.g., patients]. In cases in which our resources are limited and many people face emergencies and some of these people are family members, friends, benefactors, the correct balance is to help loved ones first. This reduces demandingness in these situations, since we are more inclined to help loved ones anyway [128].

Ackeren and Sticker’s reading of Kant’s system of duties, however, warrants criticism. For, the obvious question here is whether this is an action motivated by inclination and not duty per se, i.e., whether this follows from one’s sentiment rather than one’s reason. If so (i.e., follows from sentiment), why should this be morally compelling? The authors have not clarified this point in any way. Nevertheless, they share the Kantian ethics of duty in the sense that the plea to an emergency is a central criterion for balancing the exercise of obligatory ends, and by the same token special obligations seem more potent than other prescriptions of duty, which eventually permits a moderation of the duty of beneficence normally performed towards patients.

In answer to the second question raised above, it can be said that absence of adequate and quality PPE, and the institutional incapacity for proper treatment have made the situation exceedingly risky for physicians. This unfortunate and insuperable condition would, if not moderated, force physicians to violate a perfect duty, i.e., a self-regarding duty to avoid self-injury^33^ [131]. Overall, from the analysis presented in this section, it is apparent that, in the standard view, Kant insists on a threshold deontology for commonplace moral perspective, but he suggests a quite specific ethic for emergencies. The implication of such a split-level ethical guideline for physicians of Bangladesh is that, irrespective of their specialization, non-specialist primary care physicians should report to duty in hospitals and treat the general set of patients in need of medical care for acute and chronic conditions; while hospital authorities must ensure that only specialized practitioners (e.g., infectious disease specialists, pulmonary care specialists) treat COVID-19 patients, subject to the availability of PPE and infection control measures and their individual assessment that allows for a plea of emergency in that unique context.

### Framing “moral dilemma”

If one poses the question of “duty to treat” vs. “right to refrain” as a problem of *moral dilemma,* then for the sake of any prospective resolution one should be clear what one means by ‘moral dilemma’. Here we appropriate a perspective outlined by Michael Shaw Perry [132], although there are other views of moral dilemma^34^ [133] [134]. Perry appeals to Wittgenstein’s concept of a philosophical problem, i.e., a problem having the form, ‘I don’t know my way about’ [135]. A moral dilemma, on Perry’s account, is a situation “in which there are two incompatible ethical duties, and thus what one ought to do or who one ought to be is over-determined because there are too many right answers. Put another way, it is underdetermined because any answer violates an ethical duty we have” [132]. Perry clarifies further: “In a moral dilemma, at least two *mutually exclusive* actions have a *clear moral rationale* for them or there is simply *no moral answer* at all. It is not that we are tempted to be unmoral but that there is a *moral conflict* regarding which course of action we ought to take…I am concerned with cases in which morality itself seems to demand two different incompatible actions” [132].

Thus, in a moral dilemma one can provide a rational basis for choosing one action while also having a conflicting rational basis for choosing another action, these rational bases then providing guidance that is expressed as a practical conclusion in the sense of “Therefore, I ought to do *x*.” But precisely here is the conflict; for, one assumes one is *thinking* correctly, that one’s *judgment* is reasonable, and that one’s judgment has led to a correct statement about what one *ought* to do. These situations of moral decision are not the usual everyday “pedestrian” dilemma (‘pedestrian’ being one category of dilemma Perry identifies that we face more or less everyday). In this situation of pedestrian moral dilemma, Perry opines, we have “conflicts between duties such that one must act against one duty in fulfilling another, but in so doing one does not forfeit deeply held ethical principles or ethical traits of character. The conflict between duties makes such cases dilemmas, while the latter limitation makes these cases pedestrian” [132].

In contrast to this commonplace type of dilemma, Perry describes the sort of moral dilemma that applies in present case, i.e., *critical* moral dilemmas. This sort of dilemma he characterizes as “excruciating and nearly impossible to resolve, but thankfully rare. They involve a forced choice that is impossible to make” [132, 133]. Further, “Critical dilemmas are conflicts between core ethical character traits—a choice arises wherein one must follow one duty in such a way that another duty is circumvented to such a degree that irreparable harm is done to one’s character (or soul, or identity, etc.)” [132].

Undoubtedly, COVID-19 has positioned many medical practitioners in precisely this situation—they don’t know their way about their multiple duties, at once both clinical and moral; and, precisely because they have a sense of moral conviction as persons and as professionals, they feel the weight of decision to the point of anticipating personal moral injury from fretful decisions they try to warrant but can do so only defeasibly. Despite having clinical protocols to help them in their evaluation of patients and in implementing therapeutic interventions, the novelty of COVID-19 disease progression is such that they do not know what will work in any given patient, even minimally. At bedside it is mostly trial and error, with loss of patient life despite intensive and closely monitored care.

In the novel emergency that the COVID-19 pandemic is, these physicians have incompatible duties as well, for which they have rational basis: (1) as they report to duty in hospital (for many, on extended time shifts) as physicians, with or without sufficient PPE, having concern for their own high risk of infection from extended contact with contagion and concern for their patients, as they do what they can for infection control in tending to overload of patients in emergency rooms and ICUs; (2) as they decide *which* patients—younger vs. older; with/without co-morbidities; pedestrian patients vs. health workers/colleagues who became infected while treating patients and then themselves require inpatient care even in extremis of severe respiratory distress and organ failure; etc.—will be allocated scarce resources (e.g., who is treated when in the ER as patients lie on stretchers brought in from ambulances; who is moved from the ER to an ICU bed; who gets high flow oxygenation; who gets piped/wall oxygenation; who gets a mobile oxygen tank; who is placed on monitors of various kinds; who gets intubation and a mechanical ventilator; etc., all decisions that are not normally compromised by high numbers of patients with urgent demand and low hospital supplies); (3) as they attend to duties to family after work hours with inordinate physical and mental exhaustion (being a wife or husband; being a mother or father; being a son or daughter to elderly parents; etc., all the while hoping they will not carry infection home, if they do go home, or otherwise making the difficult choice of separating themselves entirely from family and home so as not to spread infection to loved ones); and so on.

These are not pedestrian or theoretical moral dilemmas; for, they arise in the extraordinary situation of global pandemic and nigh-total emergency and precarious conditions of life where the usual social behavior is constrained and restrained. These are dilemmas extraordinary inasmuch as they challenge who physicians are as persons and as they relate themselves as medical practitioners to their sense of what matters to them in an upright moral existence. In short, these medical practitioners understand they have multiple duties to patients, to their colleagues, to the public health at large, to their families at home, etc., and they *feel* with extraordinary stress the conflict and incompatibility of answers about duties that they give themselves. And, they know all along in the immediacy of their reflection on this scene of contention that there is no surety as they face a lawless virus that causes a bewildering pathophysiology they have not seen before in patients normally presented to their specialties. Hence, they expect that whatever they “decide” to do under the press of circumstance is entirely provisional and may cause them to fail in one or another duty.

Problematic in Perry’s description of critical dilemmas is his claim that these “provide cases wherein *no moral system* is capable of resolving the dilemma and the individual must ultimately choose *without basis*” [132]. By ‘moral system’ one can identify here the usual moral frameworks of moral theology, eudaemonist virtue ethics, act or rule utilitarianism, deontology and care ethics. The difficulty here seems to arise precisely because the force of circumstances is not a problem of conflict of moral principles but instead a conflict of perceived “essential duties.” So, if a decision is to be had, if a choice is to be made, it is presumably to be had without appeal to principle, maxim, or precedent.

But, what can that mean in the case of the medical practitioner’s critical dilemmas characterized above that portend personal moral injury whatever the decision taken? Perry comments in view of a Sartrean existential choice, which he rejects also: “In these moments, we realize our freedom and have a chance at authenticity through adopting a mode of being that is wholly our own, unbounded by any outside constraint. So even though critical moral dilemmas are perplexing and frightening, they lead us to authenticity by leading us to the situation of absolute freedom. These dilemmas are not resolved by a principle but by individual fiat” [132]. This Sartrean approach to moral decision fails in leaving one to seemingly arbitrary choice without moral fortitude, Perry argues. We can concur; for, one presumes the medical practitioner to be motivated sincerely by perceived duty despite the conflicts at hand, and to have moral fortitude relative to one or another duty s/he finds compelling. Nevertheless, Perry considers, “It may be the case that morality breaks down in critical dilemmas and this reveals something important about our moral condition, even though morality does quite well in most situations and is just as constraining as before” [132].

To avoid this perception or conclusion, Perry turns to a concept of identity to ground moral decision in the case of moral dilemma. Thus, he proposes, “Identity is complex because it is constituted by many roles” [132]. Clearly, professional roles contribute to personal identity: “Profession obviously shapes who one is and how one ought to be and act” [132]. This is certainly so for one who chose to become or is a medical practitioner. She understands herself to have duties accordingly. But, a physician may have chosen also to be a husband or wife or parent, etc., thus committing her/himself simultaneously to duties normally associated with those “familial” and associated “social” roles. Thus, Perry is correct to remind that roles are at once “factual and normative,” since “occupying these roles means being bound by certain norms” [132]. The pertinent point here is that because roles are both factual and normative, Perry argues (correctly, we opine), “This permits grounding of legitimate norms without falling into the naturalistic fallacy,” i.e., “the role itself has normative import” [132].

To say the role itself has normative import is to recognize the role to be essential to one’s self-identity. Being a physician is one such role. To follow the norms of being a physician, from whatever source, is deemed essential to such identity. And, for the most part, i.e., in the normal course of professional practice, a physician willingly and readily performs accordingly. But, a critical moral dilemma upends what is normal, even to the point of forcing a significant alteration in conduct or abandoning the role altogether. Consider a physician^35^ [85] who is a specialist in emergency medicine and experienced in critical care protocols of pulmonary medicine, working at a metropolitan hospital in New York under conditions of overload of ER and ICU, bewildered by the lawless disease progression he observes in his patients, questioning the validity of extant clinical protocols for acute respiratory distress. And, because he cannot practice contrary to, or otherwise innovate in, a therapeutic procedure he decides to abandon ICU duty entirely and limits his practice entirely to the ER where he is faced with the full press of COVID-19 patients.

This physician had a difficult decision to make, since it involves both the clinical and moral perception of a critical dilemma concerning his essential professional role as a physician having duties in the ICU, where (as Perry would put it) “the stakes are high” [132]. Was he morally right? Was he morally wrong? How does one external to the scene of immediate contention of roles judge the decision taken? Following Perry, we would have to say as he does that in critical moral dilemmas, “Right and wrong do not disappear” [132]—i.e., the moral norms remain in view. Might this physician have decided differently? Yes, he could have decided simply to continue with his duties in the ICU despite his reasonably defensible reservations about the treatment protocols operative there and then (thus in consideration of the duty of non-maleficence). Would he have been morally wrong to do so? Yes, in his judgment, he would have been wrong to persist in following the assigned institutionally mandated protocol, hence his decision.

One question is whether such a choice binds the medical practitioner in the future. The same question faces the medical practitioner in Bangladesh who chooses not to report to duty or refuses to treat patients suspected to have COVID-19, even in a hospital designated to receive this category of patients. Normally, s/he would engage and manage a patient presenting for care. But, the situation of high risk of infection in the absence of PPE and other infection control measures is precarious, a duty to self and family manifestly evident in the conflict, hence the physician’s reluctance to perform according to a professional duty that is recognized even as it is not dismissed out of hand. Reasoning in parity with Perry’s analysis here, again, we would have to say that the physician has done nothing wrong initially, having his or her appointment as medical practitioner at the hospital designated by the government to treat COVID-19 patients. S/he was not consulted in this designation of the hospital; it was forced upon him and her. From the government’s judgment, to choose not to report to duty or to refuse to treat these patients is manifest negligence of duty. Suspension from the privilege of medical practice is one consequence of that decision, in which case the physician is forced to abandon this role that is essential to his/her personal identity.

Is the government’s decision morally right? Is it morally wrong? The consequences are plain to see, whether anticipated or otherwise evaluated as “cruel fate” rather than injustice^36^ [132]. A decision is made, one way or the other. To say someone here is morally blameworthy or otherwise morally praiseworthy requires stipulation of a moral warrant for judgment that is not present in the situation of epidemic or pandemic. Aware of the conflict in essential duties proper to his or her personal identity, the medical practitioner surely feels a “moral angst” (to use Perry’s term [132]) whatever the decision taken; and it is that angst that, in more settled time of reflection, may present itself as an abiding moral injury.

Perry argues that in the face of critical moral dilemmas, “Abstractly there is no one final answer…[The] individual must approach the situation with an appreciation of the dilemma and make a choice based on his identity and the particularities of the situation. On an individual level there may very well be right and wrong answers” [132]. The physician practicing in New York made his decision and took it be morally correct, despite being torn between remaining in an overwhelmed ICU or withdrawing and turning to duty in the similarly overwhelmed ER. The physicians in Bangladesh likewise made their decisions and took these to be morally correct, despite being torn between conflicting demands, i.e., understanding what a physician must normally do but simultaneously reticent in the face of an undetermined but clearly present personal risk of infection.

Epidemics and pandemics do not present physicians with the normal course of events in professional practice. They challenge the professional role and personal identity, as these cases illustrate. Given the inordinate personal and professional stressors a medical practitioner is forced to face in situations of epidemic and pandemic emergency, institutional responses should be compassionate and forgiving (especially when hospital administrative authorities are culpable for lack of PPE and infection control) rather than simply resorting to the usual claim of negligence of duty and, therefore, suspending the physician from all further duties. In the same way the New York physician shifted from ICU duty to ER duty, the physicians in Bangladesh could have been re-assigned to other clinical duties in the same hospital facility or another, thus not diminishing the medical capacity that is already in short supply in the country. At the same time, clinics and hospitals ought not, out of duty to the routine presentation of patients, demand certificates of negative COVID-19 test, since this unreasonably and harmfully postpones essential medical care.

## Concluding recommendations

The above analyses have identified several “propositions” that, taken together, are to be considered as recommended ethical guidelines for physicians and hospital authorities during pandemic conditions of medical practice. These guidelines can be considered mostly applicable in poorly-resourced developing countries such as Bangladesh, but they can be pertinent to developed country guidelines where the same principal issues of PPE supply and adequacy of infection control measures are present as physicians respond to an extraordinary surge in patient load. Further, these guidelines may hold also in general in the case of any future highly pathogenic disease outbreak that moves in the direction of epidemic and/or pandemic conditions of medical practice.

1. During a pandemic both physicians and hospital authorities must acknowledge that physicians are placed in novel moral dilemmas that involve conflicting obligations, in which case individual physician autonomous judgment as to disposition of these dilemmas must be respected to the point of allowing reasonable deference to this judgment. (By ‘reasonable deference’ one means that it is reasonable to defer to the individual physician’s combined clinical and moral judgment, where s/he explicitly and appropriately accounts for institutional operational constraints and restraints as well as personal factors weighing upon his/her medical practice in that specific clinical setting.)
2. If the physician’s claim is that there is a professional right to refrain from the duty to treat, then it should be exercised only on a bona fide plea of extraordinary conditions of medical practice, i.e., a plea of *moral emergency* (in Kant’s sense presented in his *Lectures on Ethics*, as reviewed above) that subverts the normal normative structures of moral decision in the clinical setting.
3. Specialization in infectious disease control, emergency medicine, intensive care and critical care medicine, and anesthesiology heighten a physician’s duty to treat COVID-19 patients, but even then this remains a conditional, not an absolute, duty when there are inadequate PPE and protocols for infection control. In contrast to these specialists, primary care physicians (general practitioners) who are lacking in that specialized training and have no familiarity with specialist protocols of practice cannot reasonably be pressed into mandatory critical care service, even in situations of epidemic or pandemic, given the higher probability of clinical error and even gross negligence that would amount to failure of due attention to a prior duty of non-maleficence, not to mention that many such physicians will very likely succumb to infection because of heightened exposure risks.
4. A physician appealing to a duty to family as weightier than a duty to treat, in Perry’s sense of conflicting obligation in a moral dilemma, may refrain from a presumptive duty to treat COVID-19 patients. (In an extended family structure context such as obtains in Bangladesh, or in a situation of single parenting, this is all the more so a reasonable appeal. Further, this proposition may hold in the case of any future highly pathogenic disease that attains to epidemic/pandemic conditions. Since these appeals are in fact ethical judgments taken by a physician in view of one or another sense of moral obligation and with reference to one normative framework that is privileged in the process of deliberation, it is not reasonable to characterize such a decision as “unethical” merely because it is presupposed that a physician has an absolute duty to treat.)
5. Precisely because the acute clinical presentation of COVID-19 is uncertain and only partly understood as the disease evolves in the global population experiencing the pandemic, therefore a physician should depend primarily on his or her professional clinical judgment in evaluating what counts as an acceptable level of personal risk in the clinical setting to which s/he is attached. Hospital administrative authorities should grant such deference to individual physician judgment, even in the presence of extant treatment protocols operative from past experience (e.g., as with SARS, severe acute respiratory syndrome) but which may be put into question by the actual course of clinical outcomes in ongoing patient care.
6. Under pandemic conditions of practice a highly contagious disease such as COVID-19 presents with an unpredictable disease course, in which case the “strict hierarchy” and “culture of silence”^37^ normally recognized by physicians within institutional settings should be altered. This should be done in recognition of a more participatory culture of individual physicians contributing to the interrogation and real-time modification of extant protocols when the clinical case presentation warrants this. The fact is that physicians do suffer moral injury from clinical decisions taken while weighing conflicting personal and professional obligations (in Perry’s sense discussed earlier) under extraordinary conditions of practice that obtain in a pandemic. It is clear that wholly novel moral dilemmas present conflicting obligations, even in the best of situations where clinical ethics training or clinical ethics consultations are available.
7. It is morally indefensible for hospital administrators or government authorities to insist that physicians provide medical care in conditions of highly contagious disease such as COVID-19 in the absence of quality-assured PPE and appropriate protocols of institutional infection control.
8. A physician in Bangladesh may, in the absence of quality-assured PPE, indeed claim a professional right of autonomous judgment to refrain from treatment of either confirmed or suspected COVID-19 patients. (The same guideline applies in the case of physician practice elsewhere and in situations of future epidemic/pandemic.)
9. Consistent with the above, during pandemic conditions of medical practice, hospital and government authorities should avoid suspension from duty or removal of licensure of physicians declining to treat patients and instead allow for re-assignment of practice as appropriate to the clinician’s level of training.
10. Notwithstanding the foregoing recommendations, in Bangladesh physicians attending to patients in emergency rooms or outpatient departments have a persistent duty (a) to provide (or otherwise arrange for another consultant, e.g., when s/he has personal health conditions that increase risk of infection) a minimum of *evaluative* and *stabilizing* medical care to patients suspected of COVID-19 infection (without requiring certificate of negative test result) and (b) where PPE and/or requisite treatment facilities (oxygenation, ICU, isolation) are lacking, to arrange for *immediate* transfer of a patient to a designated COVID-19 hospital, with both *authorization* and *assurance* of admission to that hospital from the national COVID-19 coordinating center for hospital admissions. This is to avoid initiating a domino effect of “serial transfer” of a patient from one hospital to another that may well have a consequence of preventable death that amounts to gross criminal negligence [136].

## Data Availability

Not applicable.

## Footnotes

1 Lee adopts a utilitarian stance, writing: “A lethal influenza pandemic will require careful application of medical ethical principles which seeks the greatest good for the greatest number.” He does say, however, allow for protocol having an adjusted standard of care, in which case “Part of the duty to treat should allow physicians to not take risk that is unreasonable that could jeopardize their life, non-infected patients and health care team members.” Further, somewhat inconsistently, he asserts: “There is no obligation for any physician to initiate treatment in an acutely ill influenza patient with chances for a successful outcome.” He concludes: “The duty to treat the influenza patient during a pandemic is primarily for experienced clinicians…If the physician lacks the level of expertise in infectious disease they have the duty to defer on treatment and triage the patient to a specialist who can competently manage such a patient.” The question is shifted, however, in the case of COVID-19, where the novelty of the disease and inexperience of infectious disease specialists and general practitioners with the pathophysiology and progression of the disease leave it entirely unclear whether there can or will be a successful outcome, and especially so given the impoverished institutional capacity in a developing country.

2 The authors referenced cite G.C. Alexander and M.K. Wynia, “Ready and willing? Physicians’ sense of preparedness for bioterrorism,” *Health Affairs*, 22 (2003):189–197.

3 For discussion of conscientious objection, see: Ishmael Bradley. 2009. Conscientious Objection in Medicine: A Moral Dilemma. Clinical Correlations. 28 May 2009. https://www.clinicalcorrelations.org/2009/05/28/conscientious-objection-in-medicine-a-moral-dilemma/. Accessed 07 August 2020; Udo Schuklenk. 2018. Conscientious objection in medicine: accommodation versus professionalism and the public good. British Medical Bulletin, 136:1, 47–56, https://academic.oup.com/bmb/article/126/1/47/4955771. Accessed 07 August 2020; Thomas D. Harter. 2019. Why Tolerate Conscientious Objections in Medicine. HEC Forum, 13 August, 10.1007/s10730-019-09381-9. Accessed 07 August 2020.]

4 It is important to bear in mind that, as Suhas Gondi et al. say, “Personal protective equipment (PPE) shortages (e.g., masks, gloves, gowns) endanger patients and health-care workers alike during the coronavirus disease 2019 (COVID-19) pandemic.”

5 In media reports in Bangladesh, even hospitals designated for COVID-19 patients may be lacking in infectious disease control expertise to provide guidance on infection control and in proper setup of ICU and isolation options.

6 The author writes: “Having to choose your own safety over offering comfort to the dying because your hospital or health care system doesn’t have enough personal protective equipment to go around inflicts moral injury.” Further, “frontline health care workers felt betrayed by institutions that made them choose between their own safety and patient well-being.”

7 According to reported statement of the additional director-general of the Directorate General of Health Services, the government has prepared only 8634 isolation beds, 329 ICU beds, and 102 dialysis beds.

8 According to report, the health ministry issued a circular to the effect that, “No patient can be refused treatment if there are treatment facilities. If the hospitals are to refer the patients to other hospitals, that has to be done only after ensuring arrangement of treatment in consultation with the Covid-19 hospital control room of the health directorate.”

9 There are media reports of hospitals refusing to treat the usual caseload due to fear of COVID-19 infection, the health minister Zahid Maleque therefore charging medical staff with “dereliction of duty.”

10 As of 11 August 2020, Bangladesh’s Institute of Epidemiology and Disease Control Research (IEDCR) [51] reports 1,287,988 tests performed, with 263,503 positive cases (20.5%), the number of deaths at 3471 (1.32% of confirmed cases). The Directorate General of Health Services (DGHS) reports the majority of confirmed cases in Dhaka Division (*n* = 82,622; 32.7%, with Dhaka City having 52,224 confirmed cases at 6.4% of Dhaka Division cases, 70% of all cases being men). That said, the IEDCR reports (on the basis of a cross-sectional study conducted with the Institute for Diarrheal Disease Research, Bangladesh) a probable 2 million Dhaka city residents positive for COVID-19.

11 JS Mill argues that no amount of general good for society would justify violations of personal liberty. Thus, as Mill suggests, violation of liberty, e.g., the rights of a physician, is not morally acceptable. This modified form of utilitarianism is called rule utilitarianism. According to this version, we should not use utilitarian principles on a case by case basis. This version of utilitarianism defends rules that bring about the best consequences for society in the long run through maintaining stability and security desired both by individuals and community.

12 According to this report, Bangladesh’s health minister stated that government hospitals have a total country-wide supply of only 10,000 oxygen cylinders. A subsequent media report represents the situation differently, with government hospitals depending entirely on cylinder-supplied oxygen (a total of 10,394 cylinders at village/upazila level, 13,745 at divisional cities, 450 at the Kurmitola General Hospital, and 123 at Kuwait Maitree Hospital, with no monitoring to assure they are filled regularly by outside vendors. Even when cylinders are supplied to individual patients, nursing staff are not monitoring flow level and medical practitioners are not attentive to rapidly changing patient symptoms. Cylinders have a capacity of 2000 l, in which case if patients are administered between 6 to 10 liters per minute, a cylinder empties within 3.5 to 5.5 h. Since COVID-19 patients have high demand for oxygenation when in respiratory distress, there is little physicians can do, hence the higher case fatality rate in Bangladesh.

13 The authors mention Dr. Carlo Urbani in Hanoi, Vietnam, who recognized SARS in early 2003, chose to provide medical care despite objection from his wife, and who died 1 month after discovery of the disease.

14 Rashna Imam is an advocate attorney of the Supreme Court of Bangladesh and managing partner of Akhtar Imam and Associates.

15 Scott Janssen [92] characterizes ‘moral injury’ as following from psychological damage due to “an inability to forgive one’s self for perceived violations of one’s moral code,” thus “moral injury is distinct in the way it alters a person’s sense of self.” Janssen cites a physician’s remarks pertinent in present context: “I am terrified. I’m seriously considering whether I can keep working as a doctor. I may be OK—I’m young and healthy—but I can’t bear the thought of infecting other patients with a disease that could kill them. And that is the risk, without proper PPE. It’s terrifying; it’s indescribable. […] And clearly, doctors are expendable. Why sacrifice us when there aren’t enough of us as it is?”

16 The authors write: “What we need is leadership willing to acknowledge the human costs and moral injury of multiple competing allegiances. We need leadership that has the courage to confront and minimize those competing demands. Physicians must be treated with respect, autonomy, and the authority to make rational, safe, evidence-based, and financially responsible decisions. Top-down authoritarian mandates on medical practice are degrading and ultimately ineffective.”

17 The rule-governed tactic to behavior leaves exposed the query of what to do when there is no precise rule to apply to a given situation. The clear response is to develop additional rules; nonetheless, as these rules are shown to be insufficient, the requirement for yet more rules ensues. A casuistic approach to ethics generally entails repetitive growth of rules.

18 The problem here is that when rules conflict with each other, we need additional rules to support the choice of what to do, hence compounding the moral judgment and task of evaluation of applicable rules.

19 A third difficulty is that obeying a set of rules may lead one to the false sense of having acted ethically. What rules one actually obeys is a nominal obligation; consequently it may not fulfill the spirit of the rules at all.

20 A rule-bound form of conduct attracts us to search for gaps, tempting us to seek ways of fulfilling procedural demands while still acting in ways the rules were envisioned to avert.

21 As to the duties of the practitioners, The World Medical Association’s International Code of Medical Ethics stipulates that a physician shall observe the principle of the Declaration of Geneva and one of the clauses of the Declaration of Geneva reads that ‘I will not permit considerations of age, disease or disability, creed, ethnic origin, gender, nationality, political affiliation, race, sexual orientation, or social standing to intervene between my duty and my patient. I make these promises solemnly, freely and upon my honour’.

22 Besides practitioners, registered health workers (e.g., nurses) are also under ethical obligation to carry out their duties towards patients. In this matter, The Canadian Nurses Association’s Code of Ethics for Registered Nurses instructs that ‘once care of a patient has been undertaken, a registered nurse has the ethical and legal responsibility to continue to provide care for the assigned period of time’.

23 The four principles to guide ethical decision-making are (i) respect for autonomy (respect people’s decision and values), (ii) beneficence (help people), (iii) non-maleficence (don’t harm people), and (iv) justice (treat like cases alike; distribute benefits and burdens fairly).

24 The phrase “duty to care” is seldom used by medical ethicists, who resort instead to more specific rules and duties to underpin obligations. From 1975 to 2004, no article has included the phrase “duty to care” in its title in the *Journal of Medical Ethics*, and the *Journal of Medicine and Philosophy* has no result under “duty” in its 25-year subject index. The vague definition of the duty to care renders its use confusing and unhelpful.

25 Robyna Irshad Khan, a consultant anesthesiologist at the Aga Khan University in Karachi, Pakistan, maintains this view.

26 As Robyna Irshad Khan explains it, a physician working in the emergency department or intensive care unit is more exposed to virulent diseases than a radiologist. When physicians go in their field of choice, they, however, are expected to know the protracted boundary of their duty. Physicians and nurses abandon certain rights enjoyed by others with the attainment of added duties and rights conferred by their professions.

27 This is the most widely cited form of categorical imperative. However, it has formulations: (i) the principle of universality; (ii) the principle of respect; and (iii) the principle of autonomy.

28 About 50 years ago, a debate about the possibility, nature, and significance of conflicts between morality and personal well-being gained momentum. The core issue in this debate — what moral theories can reasonably demand from agents — engenders controversy about the limits of moral obligation.

29 The demandingness objection is a common argument raised against utilitarianism and other consequentialist ethical theories. For example, if our resources maximize utility through charitable contributions rather than spending them on ourselves, we are, according to utilitarianism, morally required to do so.

30 Kant’s *Lectures on Ethics*, which were lecture notes taken by three of his students on the courses he gave in moral philosophy, also include relevant material for understanding his views. The foundational doctrines of the *Groundwork* are considered as the standard approach to Kantian ethics of duty. In recent years some scholars have become dissatisfied with this standard approach to Kant’s views and have turned their attention to the later works, which includes *Lectures on Ethics*. Here Kant introduces the idea of special ethics for emergency.

31 Perfect duties are typically negative (e.g., do not lie, do not kill yourself; see *Lectures on Ethics*, VI: 419.15–8, i.e., don’t do something. For example, we have a perfect duty not to murder. This means that we must never murder under any circumstance and the opposite of a perfect duty cannot become a universal law.

32 Imperfect duties reflect the nature of human rational existence. We are born weak and frail, we cannot do everything by ourselves, and we die. These realities create interesting non-rational features of our reality: I needed someone to feed me when I was a baby. I need someone to help me when my car is stuck. I need a surgeon when my liver fails. These needs are not universal either in time or duration nor are they purely rational laws. To make these desires moral, Kant needs us to universalize them. Thus, we transform I need help at times into every [limited] rational creature has a duty to help other rational creatures at times. Thus, I have a requirement to aid others at times reflective of my own need for help at other times. This is one of the two imperfect duties for Kant. The second imperfect duty is to perfect myself. This duty arises because when I need help, I need experts. Thus, the only way that rational creatures can have their needs met is if rational creatures are developing their talents. So, I too have a need to develop my talents in order to create a universalizable rule that would make it so aid is available when I need it of sufficient ability. For more see Kant’s provisional distinction in the *Groundwork* (IV.421.fn.), and the definite one in his *Metaphysics of Morals* (VI: 390).

33 Salles and Gold [131] write: “Most physicians are trained to put patients first. That ethos is drilled into us from the time we start medical school. Indeed, we have forsaken ourselves so frequently and for so long that the medical workforce suffers from burnout at higher rates than many other professions. And, while 80% of physicians say practicing self-care is ‘very important’ to them, barely more than half of them practice it often. In other words, we do not practice what we preach.”

34 For Karen Allen [133], e.g., “there are three conditions that must be present for situations to be considered moral dilemmas. First, the person or the agent of a moral action is obliged to make a decision about which course of action is best. Here, the moral agent must choose the best option and act accordingly. Second, there must be different courses of action to choose from. Hence, there must be two or more conflicting options to choose from for moral dilemmas to occur. And third, no matter what course of action is taken, some moral principles are always compromised.” This implies that there is no perfect solution to the problem in question. Benjamin Labastin’s view [134] is supportive of it. For him, the moral agent, in moral dilemmas, “seems fated to commit something wrong which implies that she is bound to morally fail because in one way or another she will fail to do something which she ought to do. In other words, by choosing one of the possible moral requirements, the person also fails on others.” There are some kinds of moral dilemmas, but the furthermost shared of them are considered into the following: (1) epistemic and ontological dilemmas, (2) self-imposed and world-imposed dilemmas, (3) obligation dilemmas and prohibition dilemmas, and (4) single agent and multi-person dilemmas.

35 This is the self-reported dilemma faced by Dr. Cameron Kyle-Siddell, board certified in emergency medicine and critical care specialist at Maimonides Medical Center in Brooklyn, New York. For similar opinion, see Gattinoni et al. [102].

36 The comment here accounts for remarks Perry makes in reference to Sophocles’ *Antigone* and Antigone’s tragic position as she is faced with the moral dilemma of duty to family and duty to state.

37 These are expressions used by Salles and Gold [131], “Health care workers aren’t just ‘heroes’…”
